# Feasibility of Concurrent Chemoradiation in Patients treated for Esophageal Carcinoma: A Single Institutional Experience

**DOI:** 10.5005/jp-journals-10018-1089

**Published:** 2014-01-22

**Authors:** Ram Abhinav Kannan

**Affiliations:** Department of Radiation Oncology, MS Ramaiah Medical College, Bengaluru, Karnataka, India

**Keywords:** Chemoradiation, Carcinoma esophagus, Tolerance.

## Abstract

**Background:**

Chemoradiation has shown superior overall survival when compared with radiation alone in esophageal carcinoma. Due to compromised nutritional status, radiation therapy in esophageal cancer patients itself is a challenge and addition of concurrent chemotherapy leads to severe side effects even when standard dose regimens are used. The tolerance of chemoradiation for carcinoma esophagus in Indian patients is still unclear. This study is an effort to know feasibility of chemoradiation in patients treated for esophageal carcinoma in our hospital.

**Materials and methods:**

A total of 47 consecutive patients of histologically proven esophageal carcinoma who were treated in MS Ramaiah Hospital were reviewed retrospectively from January to August 2013. Out of 47 patients, 20 patients were treated with concurrent chemoradiotherapy. Patients were assessed for number of days of treatment interruptions in radiation schedule and number of planned ***vs***executed cycles of chemotherapy.

**Results:**

Out of the 20 patients treated with concurrent chemoradiotherapy, 13 patients were male (65%) and 7 (35%) were female. Median age of patients was 60 years (25-75 years). Squamous cell carcinoma was noted in majority of cases (19/20 cases). A total of 18 of 20 patients completed the planned dose of radiation and only 3/20 patients completed all planned cycles of chemotherapy. Mean number of days of interruption in radiation schedule in patients receiving chemoradiotherapy was 4.4 days.

**Conclusion:**

There was poor tolerance to chemoradiotherapy leading to reduction in the number of executed chemotherapy cycles as opposed to planned cycles, although there were no significant interruptions in radiation treatment.

**How to cite this article:** Kannan RA. Feasibility of Concurrent Chemoradiation in Patients treated for Esophageal Carcinoma: A Single Institutional Experience. Euroasian J Hepato-Gastroenterol 2014;4(1):11-13.

## INTRODUCTION

Esophageal carcinoma is one of the commonly occurring cancers in India accounting for 7.4% of male and 5.9% of female cancers.^1^ Radiation therapy has become a cornerstone of treatment for most of esophageal cancer patients. Chemoradiation has shown a superior overall survival when compared with radiation alone in esophageal carcinoma.^2^ Currently, definitive chemoradiotherapy (CRT) based on the 5-fluorouracilcisplatin (5FU-CDDP) regimen has been considered for curative intent in locally advanced or inoperable nonmetastatic esophageal cancer. Radiation therapy in patients with esophageal cancer is a challenge, because these patients have compromised nutritional status. Addition of concurrent chemotherapy in these nutritionally compromised patients leads to severe side effects even when standard dose regimens are used. This leads to treatment interruptions, treatment discontinuation or even death. This study is an effort to assess the feasibility of chemoradiation among patients treated for esophageal cancer with curative intent in our institution.

## MATERIALS AND METHODS

A total of 47 consecutive patients of histologically proven esophageal carcinoma who were treated in MS Ramaiah Medical College were reviewed retrospectively from January to August 2013. Out of 47 patients, 28 patients were treated with curative intent. Eight patients received radiation therapy alone. Twenty patients who received concurrent chemoradiotherapy are the subjects of this study. Patients treated with palliative intent or radical radiotherapy alone and those patients who had a Karnofsky performance score < 70 at the time of diagnosis were excluded from the study. All patients were treated with external beam radiation therapy to a dose of 50 to 56 Gy in 28 to 30 fractions according to institutional protocol. The choice of chemotherapy agent was based on age, nutritional status, socioeconomic status and presence or absence of comorbities. The number of days of interruptions in radiation schedule and the number of planned versus executed cycles of chemotherapy were determined.

## RESULTS

Out of the 20 patients treated with concurrent chemo-radi-otherapy, 13 patients were male (65%) and 7 patients were female (35%). Median age of patients was 60 years (25-75 years). Squamous cell carcinoma was noted in 19/20 cases (95%). Adenocarcinoma was noted in 1/20 cases. The size of lesions was ≤ 5 cm, 6 to 10 cm, and >10 cm in 9, 10 and 1 patients.

Only 18/20 patients completed the planned dose of radiation. Among patients who did not complete the planned dose of radiation, one patient developed increasing dysphagia along with raised serum creatinine value and underwent endo-scopic stent placement. Other patient developed grade IV neutropenia and defaulted further treatment.

Mean number of days of interruption in radiation schedule in patients receiving chemoradiotherapy was 4.4 days. Median was 2 days (0-20 days). Two patients had more than 7 days of interruption in radiation schedule. The reasons were an attack of myocardial infarction during the course of radiation therapy (19 days) and patient defaulting the treatment (20 days), respectively.

**Graph 1:**
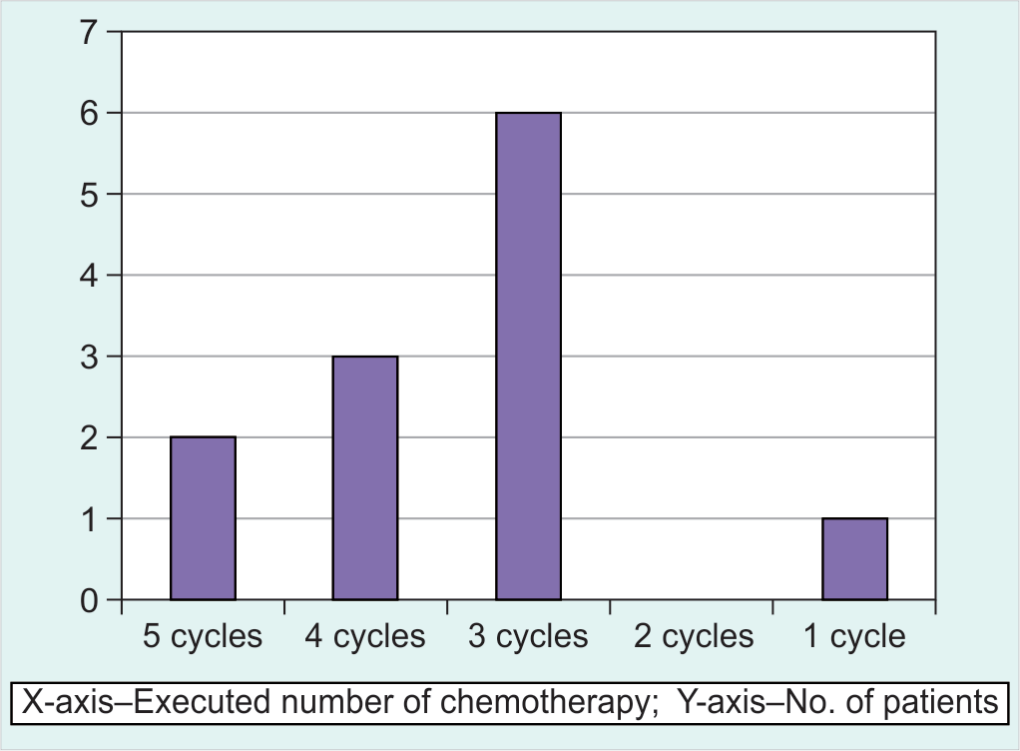
Patients planned for weekly single agent chemotherapy (Planned 5 cycles)

Out of the 20 patients who received concurrent chemotherapy, 12 patients received single agent chemotherapy and 8 patients received double agent chemotherapy. Only 3/20 patients completed all planned cycles of chemotherapy. In patients who received single agent chemotherapy, only 2/12 patients received all planned cycles of chemotherapy (Graph 1).

**Graph 2:**
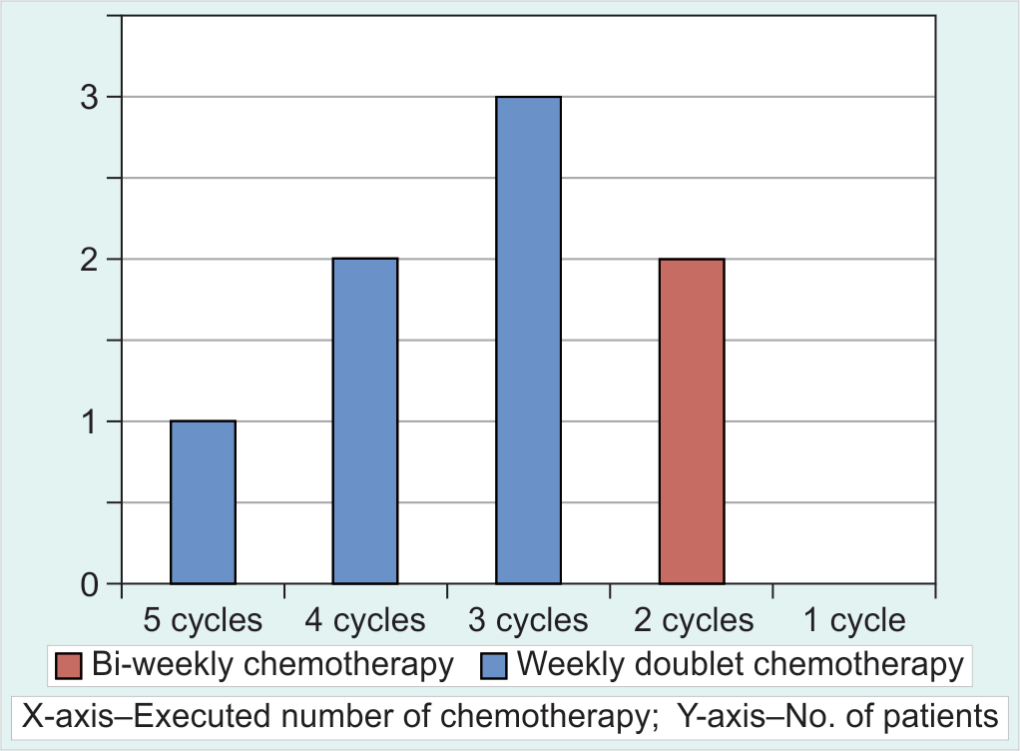
Patients planned for doublet chemotherapy (5 cycles planned for weekly regimens and 3 cycles planned for bi-weekly regimens)

In patients who received doublet chemotherapy, 1/8 completed all planned cycles of chemotherapy (Graph 2).

The reasons for reduction in planned number of chemotherapy cycles were esophagitis leading to nutritional compromise in 15 patients, myelosupression causing grade 4 neutropenia in 1 patient, raised serum creatinine in 1 patient. In these patients, chemotherapy was deferred and radiation therapy was continued.

## DISCUSSION

Definitive chemoradiotherapy is considered as the standard nonsurgical curative treatment for patients with esophageal cancers.^2^ But the addition of chemotherapy along radiation therapy has resulted in significant increase in toxicities. In a study by Herskovic et al,^2^ acute severe and life-threatening toxicities were 44 and 20% respectively in the combined therapy arm as opposed to 25 and 3% in the radiation alone arm. Similarly in a study done by Al-Sarraf et al^3^ has shown higher weight loss, myelosupression and renal toxicities in the combined therapy arm as compared with RT alone arm.

Also, the tolerance of these high-dose chemotherapy regimens in Indian patients is unclear. In the present study, only 10% of the patients were able to complete the planned number of chemotherapy cycles suggesting poor tolerance to combined modality treatment in our patients. In a study done by Smith et al^4^ 59 patients received concurrent chemoradiotherapy, only five patients did not complete the planned cycles of chemotherapy. It is important to note that only T1, T2 lesions were included in that study and supportive nutritional care, oral as well as intravenous was provided and the dose of chemotherapy in the subsequent cycles was modified based on hematological toxicities.

Loco-regional recurrence is the most common mode of failure in patients treated with combined modality treat-ment.^2^ Study done by Nishimura et al^5^ has shown that there is a 2.3% loss of local control per day increase in the treatment duration. Addition of chemotherapy along with radiation causes increased toxicites often leading to treatment interruptions in radiation schedule, which could further compromise local control.

The limitations of this study are that it is a retrospective study, sample size is small and acute toxicities need to be documented better.

In our study, there was a poor tolerance to chemora-diotherapy leading to reduction in the number of executed chemotherapy cycles as opposed to planned cycles, although there was no significant interruption in radiation treatment. There is probably a need to tailor the regimens to suit our constitutional make-up. This may lead to better compliance and thereby better control rates. Further studies should also look into effect of prophylactic feeding procedure on tolerance of chemoradiation; because, esophagitis leading to nutritional compromise was the leading cause of discontinuation of chemotherapy in our study.
